# Targeted delivery of silibinin via magnetic niosomal nanoparticles: potential application in treatment of colon cancer cells

**DOI:** 10.3389/fphar.2023.1174120

**Published:** 2023-06-27

**Authors:** Golchin Shafiei, Davoud Jafari-Gharabaghlou, Mahdi Farhoudi-Sefidan-Jadid, Effat Alizadeh, Marziyeh Fathi, Nosratollah Zarghami

**Affiliations:** ^1^ Department of Medical Biotechnology, Faculty of Advanced Medical Sciences, Tabriz University of Medical Sciences, Tabriz, Iran; ^2^ Research Center for Pharmaceutical Nanotechnology, Biomedicine Institute, Tabriz University of Medical Sciences, Tabriz, Iran; ^3^ Department of Clinical Biochemistry and Laboratory Medicine, Faculty of Medicine, Tabriz University of Medical Sciences, Tabriz, Iran; ^4^ Department of Medical Biochemistry, Faculty of Medicine, Istanbul Aydin University, Istanbul, Turkey

**Keywords:** silibinin, niosome, targeted therapy, magnetic nanoparticle, drug delivery, colon cancer

## Abstract

**Introduction:** In recent years, various nanoparticles (NPs) have been discovered and synthesized for the targeted therapy of cancer cells. Targeted delivery increases the local concentration of therapeutics and minimizes side effects. Therefore, NPs-mediated targeted drug delivery systems have become a promising approach for the treatment of various cancers. As a result, in the current study, we aimed to design silibinin-loaded magnetic niosomes nanoparticles (MNNPs) and investigate their cytotoxicity property in colorectal cancer cell treatment.

**Methods:** MNPs ferrofluids were prepared and encapsulated into niosomes (NIOs) by the thin film hydration method. Afterward, the morphology, size, and chemical structure of the synthesized MNNPs were evaluated using the TEM, DLS, and FT-IR techniques, respectively.

**Results and Discussion:** The distribution number of MNNPs was obtained at about 50 nm and 70 nm with a surface charge of −19.0 mV by TEM and DLS analysis, respectively. Silibinin loading efficiency in NIOs was about 90%, and the drug release pattern showed a controlled release with a maximum amount of about 49% and 70%, within 4 h in pH = 7.4 and pH = 5.8, respectively. To investigate the cytotoxicity effect, HT-29 cells were treated with the various concentration of the drugs for 24 and 48 h and evaluated by the MTT as well as flow cytometry assays. Obtained results demonstrated promoted cell cytotoxicity of silibinin-loaded MNNPs (5-fold decrease in cell viability) compared to pure silibinin (3-fold decrease in cell viability) while had no significant cytotoxic effect on HEK-293 (normal cell line) cells, and the cellular uptake level of MNNPs by the HT-29 cell line was enhanced compared to the control group. In conclusion, silibinin-loaded MNNPs complex can be considered as an efficient treatment approach for colorectal cancer cells.

## Introduction

Generally, cancer is a disorder in which abnormal cells are reinforced by escaping the conventional regulations of cellular division (Soltanian and Matin, 2011). Millions of people are being suffered from cancer, and the mortality caused by different kinds of cancer is dramatically growing in the world. Only in the United States, 1.7 million new cases of cancer were reported and over half a million people died of cancer in 2019, according to the Center for Disease Control (CDC) report (Hulvat, 2020). By 2029, it is estimated that there will be 16.8 million cancer deaths and 25.5 million new cancer cases annually (Thun et al., 2010). Colorectal cancer (CRC) is one of the most common malignancies in the digestive system, and it has been reported as the third most common malignant tumor worldwide (OLeary et al., 2018). The occurrence of CRC is expanding in metropolitan regions and industrialized nations, as well as nations encountering financial change, like Eastern Europe, most Asian nations, and a few South American nations (Bray et al., 2018). According to a recent study in Iran, the prevalence of CRC caused by adenomatous polyps (the most common cause) is about 34%, which is almost equal to the rate reported in developed countries. Targeted drug delivery for the pathogenesis factors of CRC has become an advance, and the recruitment of nanotechnology approaches has opened new horizons in this area (Tiwari et al., 2020).

**FIGURE 1:**
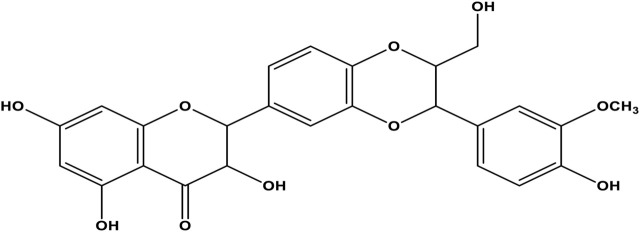
Molecular structure of silibinin.

Each cancer treatment method has benefits and disadvantages, and combined treatment is vital to get the best results (Mohammadian et al., 2016a; Mokhtari et al., 2017; Hassani et al., 2022). Since more than 85% of human cancers are solid tumors, current cancer therapy techniques usually contain invasive procedures, like chemotherapy, to shrink tumors before surgical removal (Rolston, 2017). Chemotherapy drugs target rapidly dividing cells, which are property of most types of cancer cells and some normal tissues. Although cytotoxic cancer drugs are highly effective, they exhibit significant adverse effects that limit the dose of chemotherapy drugs (Pérez-Herrero and Fernández-Medarde, 2015; Alagheband et al., 2022). Problems associated with chemotherapy are drug non-specificity caused by poor drug delivery systems. These problems are being solved using nanoparticles (NPs) as drug delivery systems (DDSs) or nanocarriers like protein cages, liposomes, micelles, niosomes (NIOs), metal, polymer, and protein NPs (Jadid et al., 2023; Jafari-Gharabaghlou et al., 2023). NIOs are a type of NPs used in drug delivery and consist of layered structures of vesicles made from non-ionic surfactants. NIOs are carriers of a hydrophobic and hydrophilic anticancer drug because they have an amphiphilic property, and can increase the half-life of the drugs conjugated with NPs in cancer cells (Coviello et al., 2015; Eatemadi et al., 2016). Owing to their lower toxicity, they improve the therapeutic index by limiting the drug action to the target cells. The non-ionic surfactant in NIOs is biodegradable and biocompatible, thus does not stimulate the body’s immune system (Liu et al., 2017). Moreover, there is no need to manage and store surfactants in a specific condition. To boost the DDS function, a localized magnetic field is applied to direct the aggregation of NIOs in the target (Liu et al., 2017). Recently, magnetic NIOs NPs (MNNPs) conjugated with anticancer drugs are investigated. Promising results are reported about stimulus-responsive drug release at the target cancer site by applying an external magnetic field (Jamshidifar et al., 2021; Maurer et al., 2021; Momekova et al., 2021; Salmani Javan et al., 2022). The role of MNPs in bio-medicine, especially in the field of drug delivery, is important because their inherent magnetism facilitates many tasks, including targeting, which is very important and necessary in drug delivery (Mou et al., 2015). The combination of NIOs and MNPs provides both carrier targeting as well as drug protection properties that can be used as an efficient system for drug delivery (Pardakhty1 and Moazeni, 2013). Metal NPs can be enclosed inside NIOs with the desired drug to form MNNPs. Within the last decades, studies have focused on magnetic delivery approaches for vesicular DDSs, however, MNNPs have not been investigated widely. Plant-based drugs are usually the extract of medicinal herbs with different therapeutic applications (Solowey et al., 2014). Within the past decades, researchers have focused on herbal extracts, because of their anticancer and anti-neoplastic properties. Silibinin is an extract obtained from the medicinal plant *Silybum marianum* (Valková et al., 2021). Silibinin is the most active component of silymarin flavonoids that induces apoptosis and in the last 2 decades has been evaluated for the treatment of many tumors (alone or in combination with other chemotherapeutic agents) (Davis-Searles et al., 2005; Kaur et al., 2009). Previously, silibinin was administrated as a supplement in foods for liver diseases remedy (Carrier et al., 2003). Today, the anticancer property of silibinin is known to be related to the presence of flavonoid factors and some strategies to fight cancer cells, including interfering with the cell cycle, inhibiting angiogenesis, invasion, and metastasis through the effect of many molecular events (Denev et al., 2020). This plant extract is an inducer of cancer cell apoptosis and has been investigated for treatment of many tumors (alone or in combination with other chemotherapeutic agents) in the last 2 decades (Ghasemali et al., 2013; Badrzadeh et al., 2014; Jahanafrooz et al., 2018). Nonetheless, hydrophobicity is the most challenging obstacle regarding the application of silibinin in the body (Figure 1) (Iyengar and Devaraj, 2020). Therefore, targeted delivery systems for silibinin are essential to minimize side effects due to systemic drug distribution and achieve a maximum therapeutic effect. This research aimed to study the role of silibinin loaded in MNNPs compared with free drugs in targeting the HT-29 colorectal cancer cell line. To this end, MNNPs and silibinin-loaded MNNPs were synthesized and their physicochemical and morphological properties were evaluated. The MTT assay was done to investigate cell viability. Furthermore, cell cytotoxicity effects, apoptosis, and the cellular uptake rate of MNNPs were examined *in vitro*.

## Materials and methods

### Materials

Fetal bovine serum (FBS) and trypsin-EDTA were purchased from Gibco (Invitrogen, United Kingdom). Silibinin, DMEM (Dulbecco’s Modified Eagle Medium), tween 80, cholesterol, sorbitan monostearate (Span 60), 3– (4,5 dimethylthiazol-2-yl)-2,5-diphenyltetrazolium bromide (MTT), trypsin, dimethyl sulfoxide (DMSO), phosphoric acid, and phosphate-buffered saline (PBS) were obtained from Sigma-Aldrich (St Louis, MO). Methanol, chloroform, ethanol, penicillin G, streptomycin, iron (III) chloride, and iron (II) chloride tetrahydrate were purchased from Merck, Germany. Annexin V-FITC apoptosis detection kit [including binding buffer, annexin V, and propidium iodide (PI)] was prepared from eBiosciences (MA, United States).

### Cell culture

The HT-29 (colorectal cancer cell line) and HEK-293 (normal cell line) were purchased from the Pasteur Institute cell bank of Iran. The cells were grown in DMEM medium supplemented with FBS (10%), 0.05 mg/mL penicillin G, and 0.08 mg/mL streptomycin. HT-29 colon cancer cells were placed in sterile flasks and incubated at 37°C in a humidified atmosphere containing 5% CO2.

### Synthesis and coating of MNPs

Generally, the co-precipitation method was applied to synthesize MNPs (Kedar et al., 1997). The amount of 6.5 g of FeCL_3_ and 4 g of FeCL_2_. 4H_2_O were blended in 100 mL of deionized water in a three-neck flask, and then 200 mL of 2 M NaOH was added dropwise into the flask and stirred for 2 h at a speed rate of 1,000 rpm under N_2_ at room temperature. The resulting MNPs were washed twice with distilled water and ethanol (96%) and the final precipitate was placed in a 50°C oven to dry. In the next step, the MNPs were coated with a succinate starch. To synthesis succinate starch, 25 mL of distilled water was added to 4 g of starch, and then 12.5 mL of 2 M NaOH was added to obtain a clear, yellowish solution. Subsequently, 3.7 g of succinic acid (SA) was added to the solution, and the mixture was stirred (1,200 rpm) for 4 h at 100°C. After cooling, 50 mL of cold ethanol (96%) was added to form a white precipitate. Afterward, the sediment was washed several times with ethanol (96%) and dried in an oven at 60°C. To prepare MNPs Ferro fluid, 100 mg of MNPs and 100 mg of succinate starch were mixed in 10 mL of distilled water and stirred (1,000 rpm) for 24 h at room temperature. Finally, the supernatant solution was separated and in order to deposit the iron nanoparticles attached with starch it was placed on a magnet, then the supernatant solution was discarded and the precipitate was collected and dried at room temperature (Fathi et al., 2014; Shahbazi et al., 2023).

### Synthesis of NIOs by thin film hydration method

Thin-film hydration method was used to synthesize NIOs. Briefly, 20 mg of Span 60, 15 mg of Tween 80, and 7 mg of cholesterol were dissolved in 6 mL of chloroform and 6 mL of methanol solvents (with a 1:1 ratio). Next, the Solvents were evaporated from the mixture by rotary evaporator (120 rpm, 60°C, 1 h). Finally, a thin layer film of milky surfactant was formed on the wall of the flask. In the next step, 10 mL PBS was added and placed in the rotary for 10 min until the transformation of pro-niosomes to NIOs (Shahbazi et al., 2023). Then, the NIOs suspension was placed in ice bath and exposed to sonication with a probe sonicator (Amplitude of 25%, 200 w) (Fisher Scientific Co., US), to reduce the size of NIOs and to break NIOs aggregates. Silibinin was added in the first step of synthesis due to its lipophilic nature while ferrofluid MNPs were added along with PBS.

### MNNPs size, surface charge, and structural characteristics

The dynamic light scattering (DLS) method was used to assess the size and surface charge of synthesized NPs. Furthermore, a transmission electron microscope (TEM) (LEO906E, Carl Zeiss, Oberkochen, Germany), operating at 80 kV was used to determine the size and morphology of the prepared MNNPs. For TEM and FT-IR analysis, the samples were freeze-dried by a freeze-dryer (Dena Vacuum, FD-5005-BT, Iran). Also, Fourier transforms infrared (FT-IR Tensor 27 spectrometer) spectrum (500–4,000 cm-1) was used to evaluate the structural analysis of synthesized NPs, and a vibrating sample magnetometer (VSM) (MDK, Iran) was used to evaluate the magnetization value of MNNPs.

### Assessment of drug loading (DL) and encapsulation efficiency (EE) of MNNPs

To determine the amount of loaded silibinin and also to remove the unloaded drug, a dialysis membrane method was performed. Briefly, 5 mL of the silibinin loaded MNNPs were added into a container with 50 mL PBS and magnetically stirred at 120 rpm. To protect the stability of MNNPs, the container was exposed to the ice. The encapsulation efficiency (EE) and drug loading capacity (DL) were determined by the measurement of unloaded drug via UV-Vis spectroscopy at 288 nm considering the calibration curve by the following formula:

$$EE\;\left(\%\right)=\frac{Mass\;of\;drug\;in\;NPs}{Total\;drug\;mass}\times 100$$

$$DL\;\left(\%\right)=\frac{Mass\;of\;drug\;in\;NPs}{Total\;NPs\;mass}\times 100$$

### Assessment of drug release rate of MNNPs

First, the MNNPs containing silibinin were transferred to two clamped dialysis bags that were inserted in distilled water for 24 h. For the simulation of normal and abnormal (cancer) conditions, PBS (pH = 7.4) and composed of methanol and 0.02 M phosphoric acid (50: 50, v/v) (pH = 5.8) were used, respectively. Buffer solutions (50 mL of each) were added into two lidded containers, and the dialysis bags were exposed to buffers. Bags were shaken (100 rpm) at 37°C. At different time intervals, 2 mL of the sample was replaced with 2 mL of fresh solution. The release pattern was calculated using UV-Vis spectrophotometer at 288 nm. Also, the release curves were evaluated by various kinetic models (Fathi et al., 2018).

### MTT assay

The cytotoxicity of drugs was evaluated by MTT assay, briefly, 6.5 × 10^3^ HT-29 and HEK-293 cells/well were seeded in a 96-well plate and incubator at 37°C with 5% CO_2_ for 24 h. Afterward, cells were exposed to various doses of free and encapsulated silibinin and incubated for 24 and 48 h. After the required incubation time, 50 µL of the MTT solution (2 mg/mL) was added to each well, and plates were covered with an aluminum foil and incubated for 4 h. After this time, the supernatant was removed, and 100 µL of DMSO was added and shaken for 10 min. Finally, the plates were transferred to the ELISA reader (Biotech Co., United States), and the optical densities (ODs) were read at a reference wavelength of 570 nm in comparison to untreated control cells.

### Cell apoptosis assessment

Flow cytometry technique was used to analyze cell apoptosis, in summary, 70,000 HT-29 cells were seeded to each well in 6-well plates. For cell treatment, silibinin and NIOs complex (40 μg/mL) were used and treated for 48 h. After separating, the cells were washed twice with PBS and centrifuged at 190 g for 5 min. Then, 100 μL of binding buffer, 5 μL of FITC (0.25 μg/mL), and 5 μL of propidium iodide (20 μg/mL) were added to each sample and left for 20 min in the dark at room temperature. Finally, 100 µL of binding buffer (0.1 M Hepes (pH 7.4), 1.4 M NaCl, and 25 mM CaCl2) was added, and reading was performed using a flow cytometry device (Thermo Fisher Scientific Co., United States) in the darkness.

### Assessment of cellular uptake of NIOs

For this purpose, 5 mg of FITC was dissolved in 1 mL of methanol, and 250 µL was added to each falcon and mixed with 1 mL of niosomal complexes. Covered falcons were placed in a shaker with ice at a speed of 100 rpm overnight, and then the contents of the falcons were washed in two steps under dark conditions using PBS. Subsequently, 70,000 HT-29 cells were seeded in each well of 6-well plates and exposed to niosomal complex for 4 h in the darkness in a humidified atmosphere containing 5% CO2. After the treatment, the cells were transferred to falcons and centrifuged for 5 min at 190 g, after discarding the supernatant, washed with PBS and centrifuged, and this process was repeated and finally, the reading was done with a flow cytometry device.

### Statistical analysis

Statistical analysis was performed using the non-parametric paired Wilcoxon test and statistical significance was described when the *p*-value was less than 0.05. All statistical analyzes were performed by Flowjo-V10 and Prism software version 9.3. The results were expressed as the mean ± standard deviation (SD) of three independent experiments.

## Results and discussion

### Measurement and characterization of synthesized MNNPs

**FIGURE 2:**
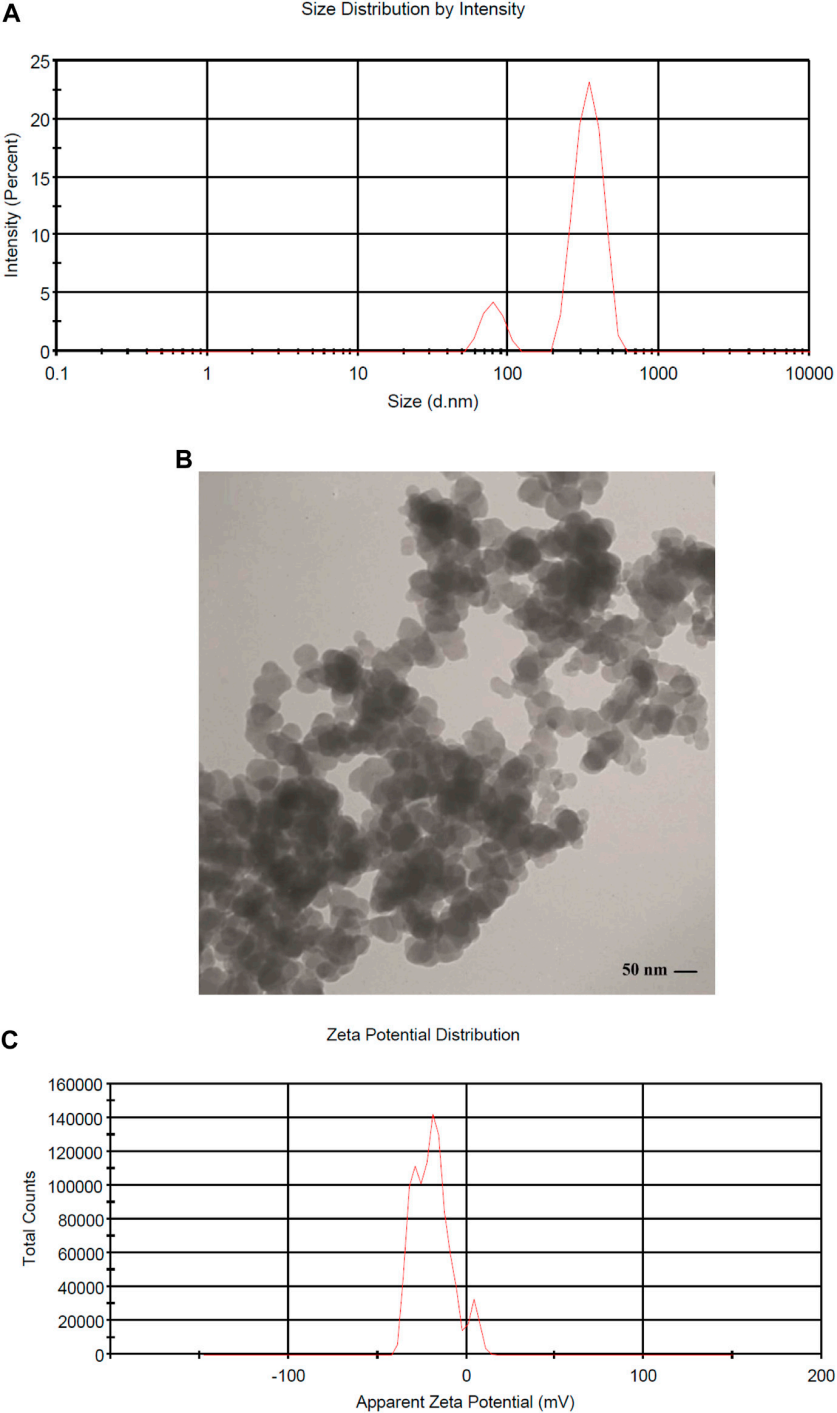
**(A)** DLS histogram showing the size distribution intensity of magnetic niosome NPs MNNPs, **(B)** Characterization of MNNPs size and morphology using Transmission Electron Microscopy (TEM), and **(C)** zeta potential distribution of MNNPs.

Generally, MNNPs size is one of the principal characteristics regarding the release of the drug from the MNNPs, physical stability, cellular absorption, and biological distribution (Barani et al., 2019). The size and surface charge of the synthesized MNNPs were checked using the DLS method. The distribution intensity of MNNPs was obtained at 70 nm with DLS (Figure 2A). According to the TEM image, shapes and distributions of MNNPs were homogeneous spherical with a size of 50 nm (Figure 2B). Typically, the size of MNNPs in the TEM images was smaller than in DLS as DLS, the hydrodynamic diameter of the MNNPs was measured. The larger MNNPs in the images are associated with the overlapping/agglomeration of some smaller MNNPs during the preparation (Liu et al., 2021). Lately, researchers have focused on the surfactants coating the MNNPs, because they can act as a steric barrier and intercept agglomeration caused by magnetic dipole–dipole attractions between MNNPs (Ramimoghadam et al., 2015). The different sizes have been reported by previous research, and it seems that other physical attributes of MNNPs like surface charge as well as different structures of chemical compounds and synthesis methods are responsible for the size of MNNPs (Barani et al., 2020; Ag Seleci et al., 2021). Considering that NPs in the range of 70–200 nm are stable in the bloodstream and are considered long-circulating agents, synthesized MNNPs with the above size can be used for smart drug delivery in pharmaceutical purposes, according to previous published studies (Barar and Omidi, 2014). In addition, it was demonstrated that NPs less than 200 nm can be escaped of the immunes system, while NPs with size less than 70 nm can be accumulated by the liver (Barar and Omidi, 2014). MNNPs charge is another important feature that has a considerable impact in entering and capturing the drug into the cell and size distribution of MNNPs.

**FIGURE 3:**
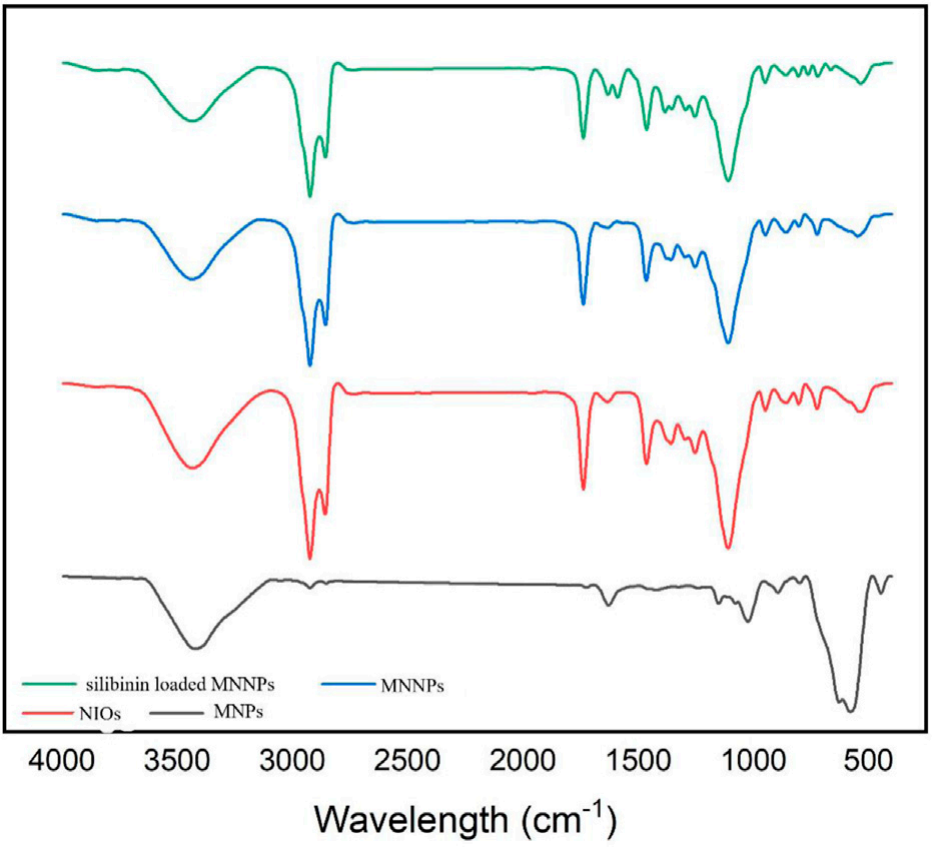
FTIR spectrum for magnetic nanoparticles (MNPs), niosomes (NIOs), magnetic niosomes nanoparticles (MNNPs), and silibinin loaded MNNPs. The results demonstrated that the silibinin was effectively encapsulated in MNNPs.

It is established that making a targeted surface charge of synthesized NPs is a practical strategy to control their assimilation into the particular destination (Osaka et al., 2009; Stiufiuc et al., 2013). Usually, a positive charge of NPs surface through coating leads to convenient and fast cellular uptake when NPs are encountered with the negative charge on the cell membrane (Su et al., 2012). In this research, the surface charge of the synthesized MNNPs was determined using the DLS method as the zeta potential was −19.0 mV (Figure 2C) and polydispersity index (PDI) was 0.52. The zeta potential result reveals that the surface charges of synthesized MNNPs are negative. This negative charge reduces the toxicity of the system and improves its performance by preventing the accumulation and deposition of NPs. FT-IR technique was used to study the chemical structure of the prepared MNNPs, and to measure the non-interaction of the synthesized niosomal system with silibinin and MNPs. Expected peaks related to MNPs, modified NIOs, MNNPs, and silibinin loaded MNNPs were monitored, which indicated that silibinin and MNPs were loaded in NIOs (Figure 3). In examining the FT-IR spectrum of drug-free NIOs (Figure 3), the peak at 3,433 cm^-1^ is characteristic of the–OH group and the peak at 1738 cm^-1^ is related to C=O stretching vibrations. Finally, the peaks at 2,923 cm^-1^ and 1,109 cm^-1^ are related to the C-H bond and vibrations of C-O bond stretching, respectively (Mehta and Jindal, 2013; Samed et al., 2018). For NIOs containing silibinin and MNPs, the peak at 1,631 cm^-1^ corresponds to C=O stretching vibrations, and the peak at 3,435 cm^-1^ is associated with the -CH bond. Furthermore, the absorption peaks at 2,923 cm^-1^, 1,108 cm^-1^, and 720 cm^-1^ belong to the C-H bond, stretching vibrations of the C-O bond, and Fe-O bond in Fe_3_O_4_, respectively (Ebrahimnezhad et al., 2013). By comparing the peaks in the FT-IR spectrum related to NIOs, MNNPs, and silibinin loaded MNNPs, it was observed that slight changes occurred in the peaks related to NIOs containing silibinin compared to NIOs without drug, and this confirmed that silibinin drug is placed in the NIOs nisome. Also, considering that no new peak was added and disappeared in the FT-IR spectrum of NIOs containing the drug, it can be assumed that no chemical interaction occurred between the NIOs and the drug as both kept their nature and remained away from change.

**FIGURE 4:**
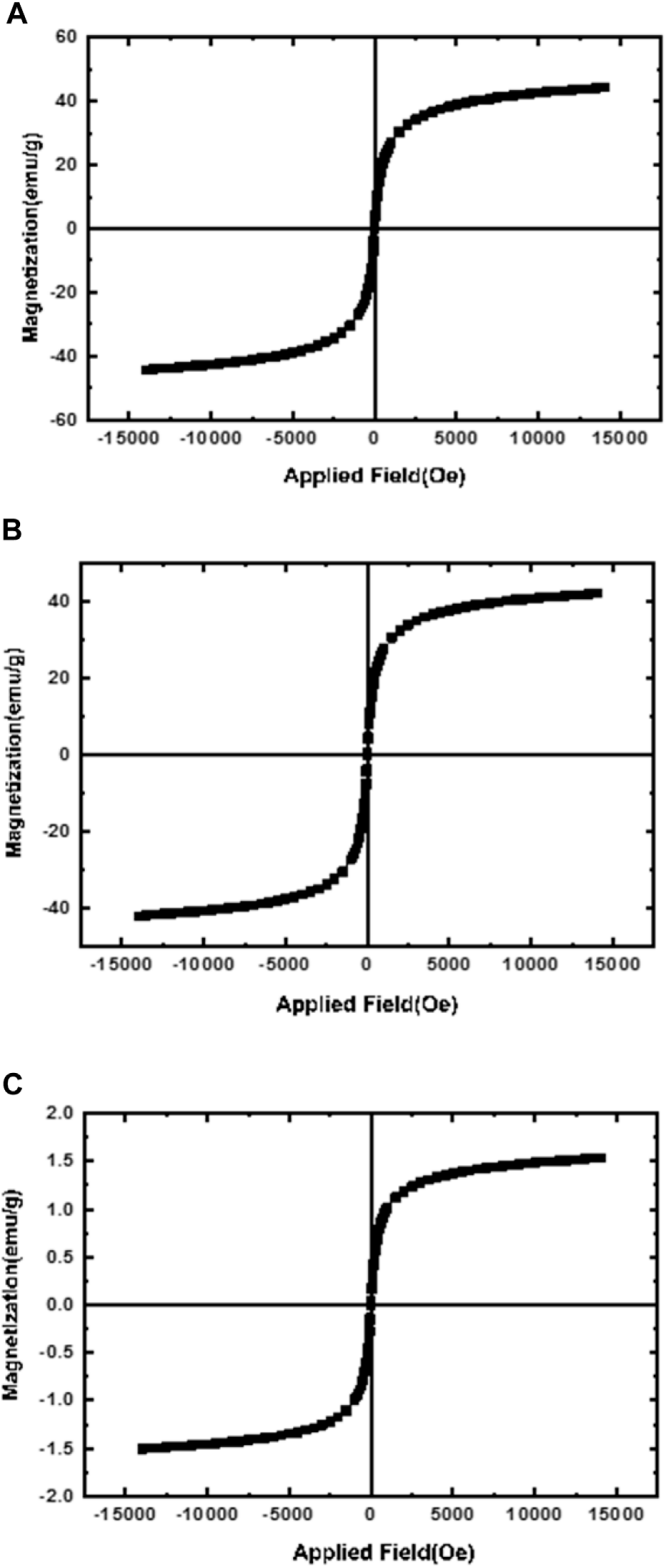
Magnetization curves of **(A)** magnetic nanoparticles (MNPs); **(B)** Starch-modified MNPs, and **(C)** magnetic niosomes nanoparticles (MNNPs). The results confirmed the loading of MNPs and silibinin in NIOs is done properly.

Vibrating sample magnetometer (VSM) was used to evaluate the superparamagnetism of NPs. Since the reason for using magnetic NPs is the targeted delivery of the carrier by applying an external magnetic field, it is very important and necessary to determine the magnetic properties of these NPs. the magnetic activities of MNNPs, starch modified MNPs, and MNPs were demonstrated using vibrating sample magnetometry (VSM) at room temperature. The hysteresis curve in Figure 4A; Figure 4B showed that the saturation magnetization of MNPs was 42 emu/g. Compared to the saturation magnetization of MNNPs in Figure 4C, which is 1.5 emu/g, this value is higher. This difference confirms that the loading of MNPs and silibinin in NIOs is done properly, according to previous studies published in this area. The Figure 4 does not show any hysteresis curve indicating the superparamagnetic behavior of the synthesized particles (Yang et al., 2006).

### Encapsulation efficiency, drug release rate of MNNPs and kinetics models

**FIGURE 5:**
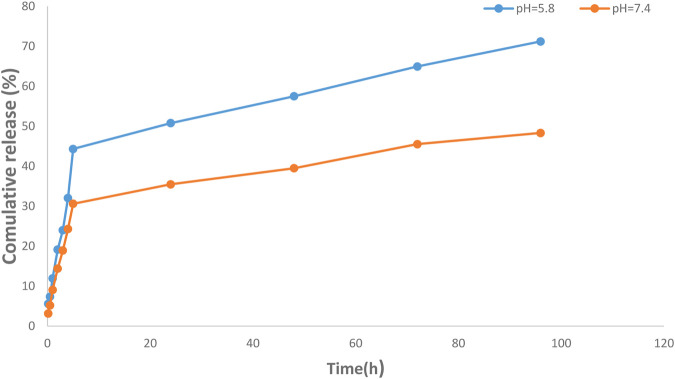
Investigation of drug release efficiency from magnetic nisome nanoparticles (MNNPs) at pH = 5.8 and 7.4 at 37°C. The pores of the dialysis membrane are large enough to allow the free movement of silibinin, but small enough to prevent the diffusion of nanoparticles. The results demonstrated a sustained release pattern from the nanoparticle with a partially burst-release at the initial stage and followed sustained during the 96 h study period. Data are presented as mean ± SD of three independent experiments.

Encapsulation efficiency (EE) is defined as the ratio of the amount of drug in the MNNPs to the total amount of drug applied in NPs formulation. Currently, a large number of NPs systems have relatively low EE, thus developing strategies to increase EE remains a challenge (Liu et al., 2019). EE has been increased while there is a drug concentration increase thanks to polymers and MNNPs, based on the previously published studies (Prabha and Raj, 2016; Ghadiri et al., 2017; Erdagi and Yildiz, 2019). Jin et al. (2018) argued lowering the pH is a promising way to boost the EE efficiency up to approximately 70% in the MNNPs formation while this efficiency without pH change is between 10% and 15% at maximum. In the present study, the EE in NIOs was more than 90%, which indicates the high potential of MNNPs in EE. Span 60 and also cholesterol which are used in the NIOs structure increase the amount of the hydrophobic drug loading into NIOs (Jadon et al., 2009). The results indicated the release of the silibinin with a gentle and slow gradient from MNNPs. Evaluating the drug release pattern shows that the designed NIOs, while indicating a controlled release, have a burst release within 4 h, then in the conditions of normal cells (pH 7.4) and cancer cells (pH 5.8) released about 49% and 70%, respectively during 100 h (Figure 5). These findings reveal that MNNPs are sensitive to different pH. The high acidity of tumor cells is attributed to hypoxia, or lack of oxygen owing to the inadequate blood supply (Justus et al., 2013). It was demonstrated that the MNNPs indicated better performance in tumor microenvironment (pH 5.0–6.8) compared to the physiological condition which make them the suitable candidate for DDS (Mohammadian et al., 2016b; Myat et al., 2022). Considering that tumor cells have a significantly acidic cytoplasmic pHs compared to normal cells, therefore MNNPs are more efficient with more DR at acidic pH compared to pH = 7.4 (AlSawaftah et al., 2022). This makes NIOs a good candidate for drug delivery in cancer. The results indicated the release of the silibinin with a gentle and slow gradient from MNNPs. Our findings are in agreement with previous studies that confirmed the promising role of MNNPs in the drug release process. Barani et al. reported, the drug’s release profile from MNNPs showed a first rapid release (about 50% of the drug was released within 10 h), followed by a slower and longer-lasting release (65% of the drug was released within 30 h) in acidic conditions (Barani et al., 2019). Similar outcomes were obtained by Zheng et al. (2009) Preparation and definition of magnetic cationic liposomes for gene transfer research. Tavano et al. (2013) synthesized MNNPs loading doxorubicin (an anticancer drug) for smart drug delivery and argued that the utilization of MNNPs can improve the release rate of formulations. Table 1 shows various kinetic models that were used to fit the silibinin release curves at different pHs. According to Table 1, the fitted model for release curves were Pawer law and Reciprocal powered time at pH = 7.4 and 5.8.

**TABLE 1:** The kinetics models used to fit the silibinin release data from MNNPs at different pHs. The bold values indicated the best fitted models.

Kinetics model	Equation	Coefficient of determination (*R* ^2^)	
		pH = 5.8	pH = 7.4
Zero order	$F=k_{0}\;t$	0.7542	0.7274
First order	$\ln\left(1-F\right)=-k_{f}\;t$	0.8673	0.7959
Higuchi	$F=k_{H}\sqrt{t}$	0.9030	0.8896
Power law	$\ln F=\ln k_{P}+P\ln t$	**0.9506**	**0.9627**
Square root of mass	$1-\sqrt{1-F}=k_{1/2}\;t$	0.8131	0.7622
Hixson-Crowell	$1-\sqrt[{3}]{1-F}=k_{1/3}\;t$	0.8319	0.7736
Three seconds’ root of mass	$1-\sqrt[{3}]{{\left(1-F\right)}^{2}}=k_{2/3}\;t$	0.7937	0.7507
Weibull	$\ln\left(-\ln\left(1-F\right)\right)=-\beta \ln t_{d}+\beta \ln t$	0.9462	0.9208
Reciprocal powered time	$\left(\frac{1}{F}-1\right)=\frac{m}{t^{b}}$	**0.9610**	**0.9365**

### Cell cytotoxicity of MNNPs

**FIGURE 6:**
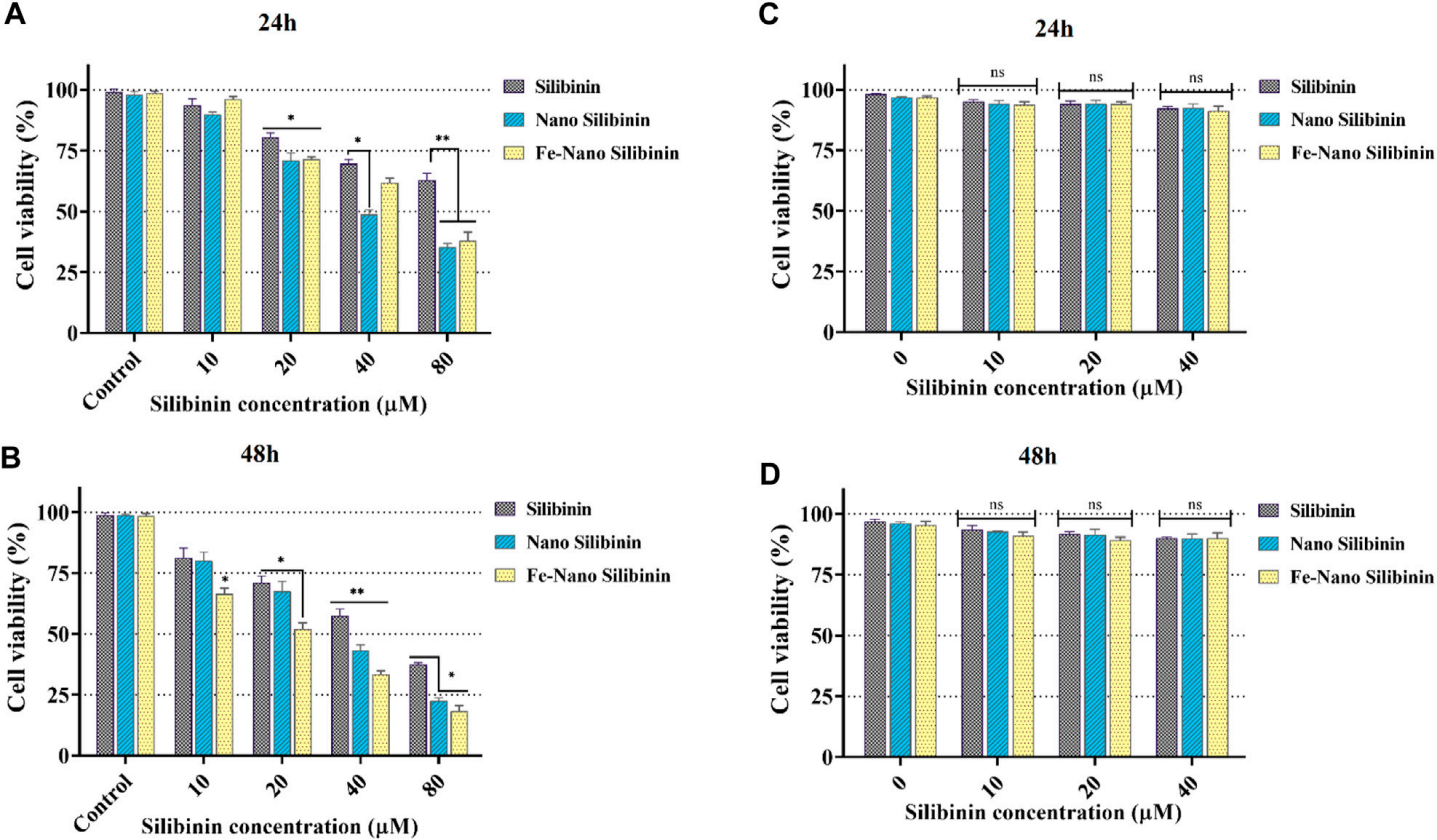
MTT assay results revealed *in vitro* cytotoxicity of blank NIOs (control), Silibinin, Silibinin loaded NIOs, Silibinin loaded magnetic NIOs NPs on HT-29 colon cancer cells [**(A)** and **(B)**], and HEK-293 normal cell line [**(C)** and **(D)**] after 24 h and 48 h incubation time. Encapsulation of silibinin in MNNPs improved its cytotoxic effect on HT-29 colon cancer cells, while it had no significant cytotoxic effect on HEK-293 cells. Data are presented as mean ± SD of three independent experiments. Error bars show standard variations and significance is indicated by *p < 0.05, **p < 0.01 and ***p < 0.001 compared to the control group.

Through receptor-mediated endocytosis, targeted NPs can enter the target cells, increasing the concentration of drug molecules within the cell. P-glycoprotein does not recognize drug molecules during this process, and membrane efflux transporters pump free drug molecules out of the cell (Wang et al., 2016). In the cell cytotoxicity assessment by the MTT method, HT-29 and HEK-293 cells were treated with free-drug and silibinin MNNPs for 24 and 48 h at various concentrations (10, 20, 40, and 80 μg/mL) (Figure 6). The results showed that NIOs containing silibinin caused more cell death in the studied times compared to pure silibinin in the HT-29 colon cancer cells and had no significant cytotoxic effect on HEK-293 cells. Thus, nanocarriers can increase the cell cytotoxicity of the drug against HT-29 cells, and there is a dose-dependent and relatively time-dependent manner regarding using complex. These results confirm the previous studies reported that the cytotoxicity of the drug-loaded MNNPs follows dose-dependent and time-dependent trends (Huang et al., 2015; Dhavale et al., 2021). The IC50 of the MNNPs complex was observed at 40 μg/mL within 48 h, therefore this concentration was considered for the following tests. The survival of over 90% of cells under drug-free NIOs treatment revealed that MNNPs have a very tiny lethal effect on the cells. This biocompatibility makes these MNNPs excellent candidates for therapeutic purposes. Additionally, the MTT test revealed that there was no significant correlation between the control group and silibinin-free NIOs.

### Apoptosis and cellular uptake analysis on HT-29 cell line

**FIGURE 7:**
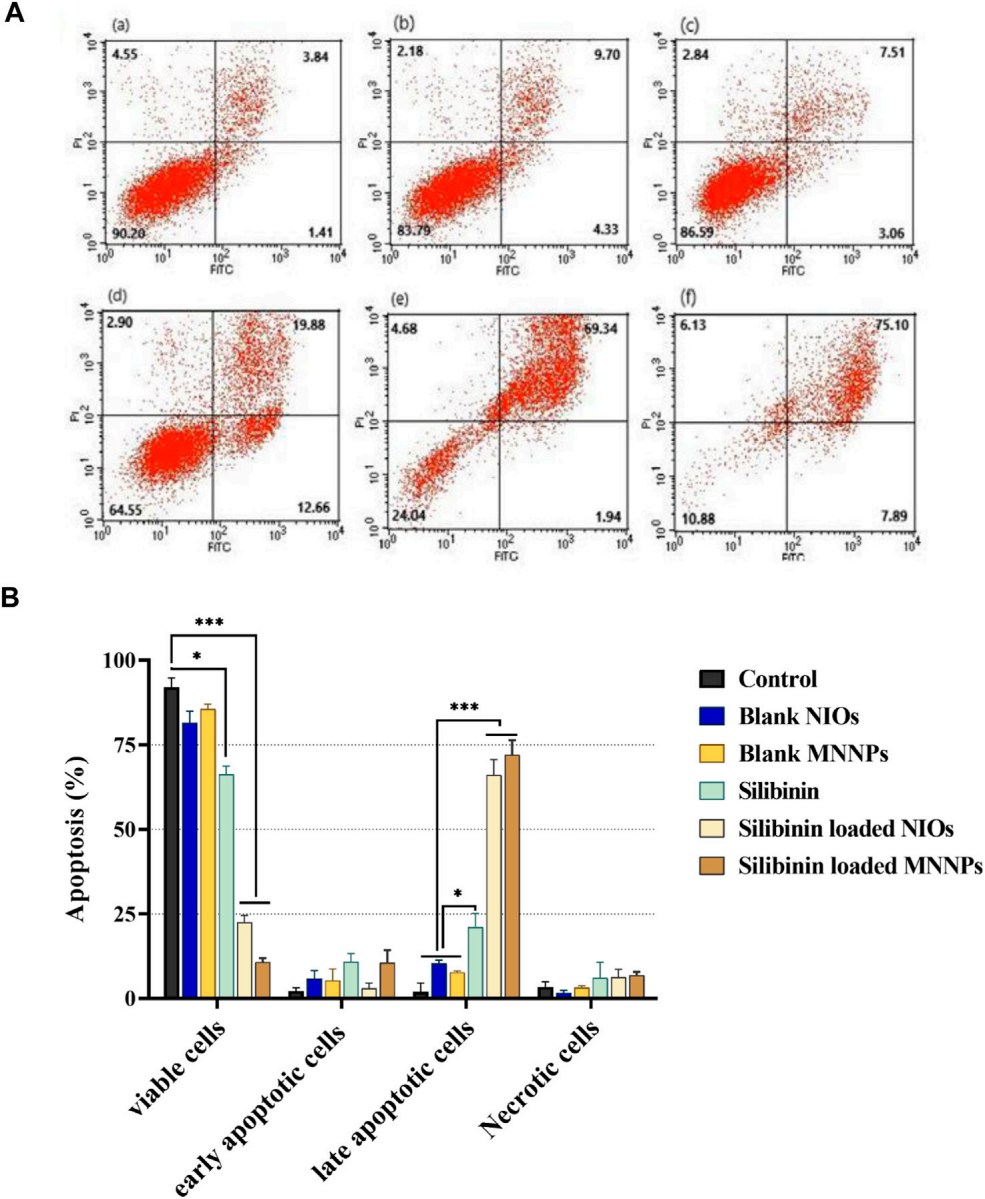
**(A)**. Apoptosis analysis on HT-29 cell line under treatment (a) control, (b) blank NIOs, (c) blank MNNPs, (d) Silibinin, (e) Silibinin loaded NIOs, (f) Silibinin loaded MNNPs. **(B)**. HT-29 cells treated with pure silibinin and niosomal complexes were evaluated after 48 h by FITC-labeled annexin V/PI flow cytometry. The obtained results confirmed that NIOs loaded with the silibinin significantly increase the apoptosis of HT-29 cells, which emphasizes the increase in the absorption of drugs from NIOs by the cells. This result is consistent with the MTT results. Data were analyzed using Flowjo-V10 analysis software and shown as mean ± SD of three independent experiments. Significance is indicated by *p < 0.05, **p < 0.01 and ***p < 0.001 compared to the control group.

HT-29 cells that treated with pure silibinin and silibinin loaded MNNPs were evaluated after 48 h' incubation by FITC-labeled Annexin V/PI flow cytometry technique. As shown in Figure 7, silibinin-loaded MNNPs significantly induce apoptosis of HT-29 cells more than other forms, furthermore, silibinin-loaded MNNPs exhibited a greater cytotoxic effect compared to the free silibinin, which demonstrates the increase in the uptake of silibinin from MNNPs by the cells. These findings are consistent with earlier research, which indicated that the internalization and subsequent intracellular release of the anticancer drug from NPs is responsible for the cytotoxic effect of NPs (Zaki, 2014). It is proposed that this formulation (niosome and magnetic niosome) can increase the drug bioavailability which can induce cancer cell apoptosis by causing DNA damage or the oxidation of proteins, lipids, and enzymes, resulting in cell death and also helping to destroy and inhibit the mitochondrial respiratory complex (Rodrigues Pereira da Silva et al., 2016; Van Houten et al., 2016; Juan et al., 2021). El-Far and co. argued that the cytotoxicity percentage by IC50 value between paclitaxel–metal NPs–NIOs and oxaliplatin–metal NPs–NIOs at the same concentrations did not differ significantly (pH 0.5). Therefore, they recommended that MNNPs are considered a suitable targeting nanocarrier system for drug delivery in colorectal cancer, target tumor cells and prolong circulation for both drugs (El-Far et al., 2022).

**FIGURE 8:**
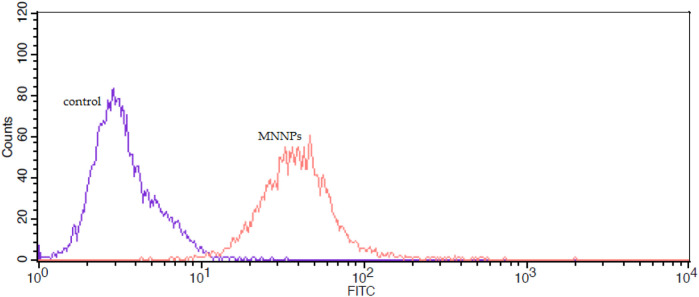
The flow cytometry analysis of uptake of FITC-labeled magnetic nisome nanoparticles (MNNPs) by HT-29 cells.

Also, cellular uptake was studied by FITC labeled NPs and via flow cytometry. Figure 8 illustrates the cellular uptake level of MNNPs by the HT-29 cell line was 99% compared to the control group. These findings indicated that the MNNPs could enhance cellular uptake significantly and induce apoptosis in the HT-29 cell line after treatment, and are consistent with previous studies that used flow cytometry to examine apoptosis and MNP uptake in cells (Depan and Misra, 2012; Li et al., 2018; Lu et al., 2018).

## Conclusion

In the current study, silibinin was loaded into NIOs containing MNPs (MNNPs) as a novel formulation to evaluate its apoptosis effect in colorectal cancer cells treatment. The size distribution, surface charge, and chemical structure of synthesized MNNPs were suitable for immune escape and confirmed that silibinin was entrapped in NIOs. According to the obtained cytotoxicity evaluations, silibinin loaded in MNNPs has more cytotoxic effects on HT-29 colon cancer cells in a dose- and time-dependent manner, and the cellular uptake of MNNPs by the HT-29 cell line was higher than controls. Drug loading evaluation exhibited a high efficiency and drug-loaded MNNPs showed an accelerated release rate in acidic pH in cancer cells compared to the neutral condition. To sum up, the MNNPs as nanocarriers are excellent candidates for the treatment of cancer cells, especially colorectal cancer. However, further *in vivo* experiments are required to prove the potential of the developed formulation as a suitable vehicle for the various cancerous cells treatment.

## Data Availability

The original contributions presented in the study are included in the article/supplementary materials, further inquiries can be directed to the corresponding author.
